# Letrozole Co-Administration in Progestin-Primed Ovarian Stimulation (PPOS) Protocols for Patients Undergoing In Vitro Fertilization: A Systematic Review

**DOI:** 10.3390/jcm15020410

**Published:** 2026-01-06

**Authors:** Raffaella Di Girolamo, Maria Giuseppina Trinchillo, Luigi Vigilante, Roberta Ordichelli, Matteo Giudice, Giuseppe Gabriele Iorio, Ida Strina, Federica Cariati, Luigi Carbone

**Affiliations:** 1 Department of Public Health, School of Medicine, University of Naples Federico II, 80131 Naples, Italy; mgtrinchillo@gmail.com (M.G.T.); federica.cariati@unina.it (F.C.); 2 Department of Neuroscience, Reproductive Sciences and Dentistry, School of Medicine, University of Naples Federico II, 80131 Naples, Italy; 3 Policlinico Federico II Hospital, University of Naples Federico II, 80131 Naples, Italy; drcarboneluigi@gmail.com

**Keywords:** POSEIDON, ovarian stimulation, letrozole, PPOS, assisted reproduction

## Abstract

**Objective:** To systematically analyze and synthesize the evidence from the literature, we compared outcomes of the PPOS + LE protocol versus standard PPOS in patients undergoing IVF. **Materials and Methods:** We systematically searched the MEDLINE, Scopus, EMBASE, and Science Citation Index databases to identify relevant studies. The clinical questions were developed according to the PICO framework. Quality assessment of the included studies was performed using the Newcastle–Ottawa Scale. Primary outcomes were ovarian stimulation outcomes (oocyte retrieved and mature oocytes). Secondary outcomes were hormonal levels during COS and pregnancy outcome. **Results:** Five retrospective studies compared oocyte yields between the PPOS and PPOS + LE protocols across diverse populations. While some authors reported significantly higher numbers of retrieved and mature oocytes with letrozole co-administration in a normal infertile population and in POSEIDON group 4, others found no benefit in the PCOS or POSEIDON 3 groups, indicating variable efficacy depending on patient characteristics. **Conclusions:** Incorporating letrozole counteracts the intense pituitary suppression typically associated with standard PPOS, increasing LH levels and the number of retrieved and mature oocytes in normal and poor responders, but not in PCOS women. Retrospective data do not allow for definitive conclusions to be drawn. Further studies are needed to confirm these results.

## 1. Introduction

Controlled ovarian stimulation (COS) is a crucial component of assisted reproductive technology (ART) [1]. Over time, the goal has been to achieve the optimal number of oocytes to enhance the likelihood of pregnancy [2]. Traditionally, standard protocols have utilized GnRH analogs (both agonists and antagonists) to avoid an early surge of luteinizing hormone (LH) [3]. Currently, new strategies that are more patient-friendly and potentially effective have led to the development of alternative protocols [4].

The Progestin-Primed Ovarian Stimulation (PPOS) protocol has emerged as a beneficial alternative, using oral progestins to prevent the LH surge induced by rising estradiol (E2) [5], although the mechanisms by which it acts remain unclear. It could be that progesterone provokes a reduction in GnRH pulse frequency [6].

This approach offers multiple benefits, including oral administration, cost-effectiveness, applicability across different patient groups based on ovarian response [7], and reduced risk of ovarian hyperstimulation syndrome (OHSS) [8,9].

Moreover, a specific consequence of the PPOS protocol is the need for a freeze-all strategy, because the early exposure of the endometrium to a progestin shifts the implantation window, rendering a fresh embryo transfer impossible; hence, the application of this protocol takes place where cycle segmentation is of choice, as, for example, in the case of preimplantation genetic testing (PGT) and Duo-stim [10].

Letrozole (LE), a third-generation aromatase inhibitor, acts by blocking estrogen synthesis from androgens [11], temporarily increasing intraovarian androgens [12], which may determine increased Follicle-Stimulating Hormone-receptor (FSH-R) expression [13] and insulin-like growth factor-1 (IGF-1) production in granulosa cells, thereby amplifying follicular sensitivity to FSH and further enhancing folliculogenesis [14,15]. In addition, a lower estradiol level reduces negative feedback on the pituitary, inducing the increased secretion of both FSH and LH [16]. Its use is explored in various contexts, including ovulation induction for polycystic ovary syndrome (PCOS) and for patients with hormonally responsive cancer aimed at fertility preservation treatments [17], as well as IVF protocols for poor-responder patients, to improve oocyte quality [18,19].

Given the advantage of the PPOS protocol, but considering that it could be associated with increased pituitary suppression and the need for gonadotrophin, and also considering the possible effects of letrozole on the ovary during ovarian stimulation, recently, letrozole has been combined with the PPOS protocol to offer synergistic advantages. In fact, it is hypothesized that letrozole could reduce the need for gonadotropins, enhance follicular response, or positively modulate the endocrine environment [20].

Despite the growing interest in PPOS and the use of letrozole as an adjuvant, it is necessary to clarify whether the PPOS plus letrozole (PPOS + LE) combination offers tangible advantages over PPOS alone.

This review, therefore, aims to systematically analyze and synthesize the evidence from the provided scientific articles, comparing the efficacy of PPOS + LE versus standard PPOS in patients undergoing IVF.

## 2. Materials and Methods

This study was exempt from institutional review board approval because it did not involve human subjects or direct intervention. We adhered to the Preferred Reporting Items for Systematic Reviews and Meta-Analyses (PRISMA) guidelines [21]. The PRISMA checklist for this review is available in the Supplementary Materials, Table S1.

We systematically searched the MEDLINE (PubMed), Scopus, EMBASE, and Science Citation Index (SCI) databases to identify relevant studies published up to October 2025.

The reference lists of relevant reviews and articles were hand-searched. Combinations of the following keywords and MeSH search terms were used: “PPOS”, “progestin primed ovarian stimulation”, “progestin primed”, “letrozole”, “aromatase inhibitors”, “IVF”, “assisted reproductive technology”, “ART”, “ovarian”, “stimulation”. No time restrictions were adopted. Only studies written in English were considered, and queries were limited to human studies. The reference lists of relevant reviews and articles were also manually searched. This review compared ovarian stimulation, hormonal levels, and pregnancy outcomes between PPOS + LE and the PPOS alone protocol in women undergoing ART. No restrictions were imposed based on age or ethnicity.

The clinical questions were developed according to the PICO framework (P = population; I = intervention; C = comparison; O = outcomes) [22,23]:

Population: Infertile women undergoing ART.

Intervention: Controlled ovarian hyperstimulation using PPOS protocol + Letrozole (PPOS + LE).

Comparison: Controlled ovarian hyperstimulation using the PPOS protocol alone.

Outcomes: Ovarian stimulation outcomes (oocyte retrieved and mature oocytes), hormonal levels during COS, and pregnancy outcomes [24]. In terms of study design, this review included both retrospective and prospective studies. Regarding publication type, we included peer-reviewed articles. We excluded non-peer-reviewed articles, preprints, reviews, opinion papers, research letters, commentaries, editorials, case studies, conference abstracts, posters, and protocols to avoid publication bias. We used EndNote X9 (Clarivate) to eliminate duplicates from the retrieved studies. Then we screened the titles and abstracts of the remaining articles. In the final phase, we evaluated the full texts of the studies shortlisted in the preceding step. Three reviewers (R.D.G., M.G.T., and L.V.) independently evaluated the titles and abstracts. Only full-text articles were considered eligible for inclusion. Disagreements were resolved by discussion among the authors and, if required, with the most experienced authors (I.S. and L.C.). Three reviewers (R.O., M.G., and G.G.I.) extracted data independently using predefined data fields and study quality indicators. Discrepancies were resolved by discussion with the senior authors (F.C., I.S., and L.C.). From each study, we extracted the author names, publication year, population, IVF protocol (if PPOS + LE or PPOS), exclusion criteria, intervention, comparison, and outcome, categorizing them according to ovarian stimulation outcomes and pregnancy outcomes. Quality assessment of the included studies was performed using the Newcastle–Ottawa Scale (NOS) [25]. According to NOS, each study is evaluated based on three main aspects: the selection of the study groups, the comparability of the groups, and the determination of the outcome of interest. Evaluating the selection of a study involves assessing how representative the exposed group is, how the non-exposed group is chosen, how exposure is confirmed, and whether the outcome was absent at the beginning of the study. The assessment of comparability includes examining whether the cohorts are comparable based on the study design or analysis. Lastly, assessing the outcome of interest involved reviewing how the outcome was measured, as well as the duration and adequacy of the follow-up. According to NOS, a study can receive up to one star for each criterion within the selection and outcome categories. A maximum of two stars can be awarded for comparability [26].

## 3. Results

### 3.1. General Characteristics of the Included Studies

**Figure 1:**
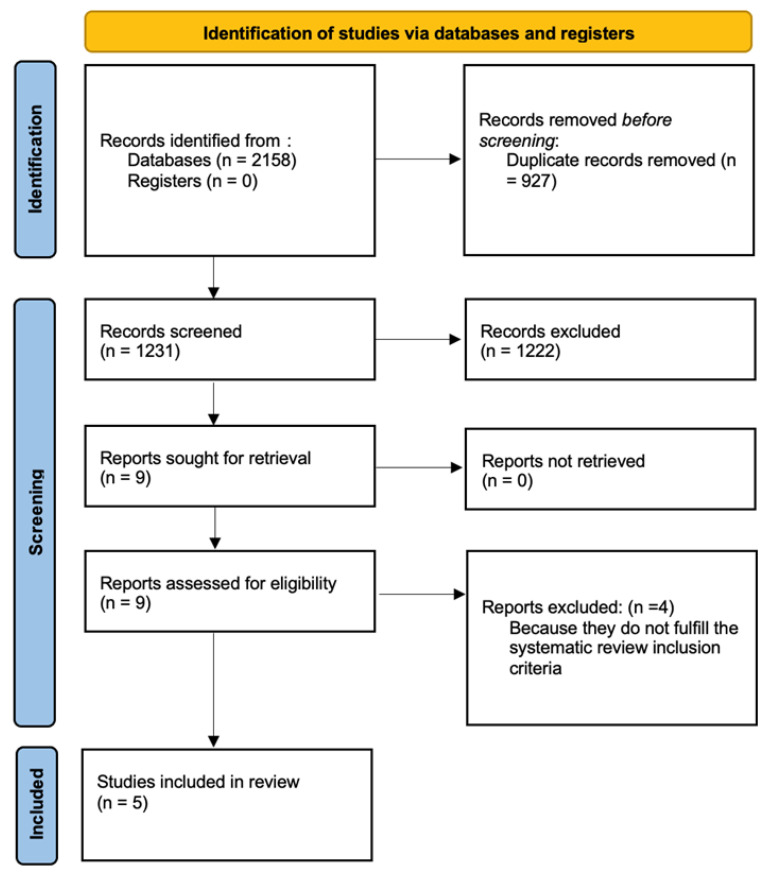
PRISMA flow diagram.

A PRISMA flow chart of the included studies is detailed in Figure 1. All the studies were conducted in China and were retrospective cohort analyses. In total, we included five studies [20,27,28,29,30]. Some of them were performed in the same centers and by the same authors, but considering different subpopulations. In detail, Jiang et al. [20] evaluated the differences between PPOS and PPOS + LE protocols, taking into consideration women with normal ovarian reserves. Liu et al. [27] observed the IVF outcomes of these ART protocols in women with PCOS while also evaluating PCOS women with high BMI [28]. Wang et al. [29] examined PPOS + LE versus PPOS alone in a general IVF population. Finally, Wang et al. [30] analyzed how these protocols could be applied to POSEIDON groups 3 and 4. Liu et al. [27,28] and Wang et al. [29] performed their analyses using propensity score matching (PSM) to reduce the selection bias of patients within their cohorts (Table 1). The risk of bias analysis is detailed in the Supplementary Materials, Table S2. We excluded Li et al. [31] because they evaluated different doses of medroxyprogesterone acetate (MPA) within the PPOS + LE protocol. Moreover, Wang et al. [32] was excluded because they took into consideration different therapy durations with letrozole within the PPOS + LE protocol. Furthermore, Zhang et al. [33] was excluded since they observed IVF outcomes within the PPOS + LE using fixed versus degressive doses of MPA. Last but not least, we excluded another study by Li et al. [34] that explored the association between letrozole and PPOS in poor ovarian responders of advanced age, which was only published as an abstract.

### 3.2. Synthesis of the Results

#### 3.2.1. Ovarian Stimulation Outcomes

All the included studies evaluated the oocytes retrieved and the mature oocytes comparing PPOS protocols with or without letrozole. Jiang et al. [20] observed an increased number of both retrieved and mature oocytes in the PPOS + LE protocol. Furthermore, Liu et al. [27] did not find the same results in PCOS patients, but actually found a trend towards increased numbers of both retrieved and mature oocytes in the PPOS alone protocol in overweight PCOS women [28]. Furthermore, Wang et al. [29], using PSM, demonstrated an increased number of retrieved and mature oocytes in the PPOS + LE protocol compared to PPOS alone. In addition, Wang et al. [30] showed that, in POSEIDON group 3, there was no difference in retrieved and mature oocytes between PPOS alone and with letrozole, while they found a statistically increased number for both outcomes in POSEIDON 4 women. When observing the duration of the stimulation and the doses of medications, Jiang et al. [20] found that ovarian stimulation lasted longer in the combined group than in the PPOS alone group, and that the total amount of gonadotrophins and MPA in PPOS + LE was significantly less than that in PPOS alone group. Liu et al. [27,28] found a similar duration of stimulation and total gonadotrophin doses between the groups in PCOS women. Moreover, Wang et al. [29] demonstrated that the days of stimulation were statistically more and that MPA doses were statistically less in PPOS + LE group, while there was no difference regarding total dose of gonadotrophins (even though a trend towards increased doses was noted in the PPOS alone group). Finally, Wang et al. [30], in both POSEIDON groups 3 and 4, did not observe differences in the duration of the stimulation and gonadotrophin doses. Interestingly, Liu et al. [27,28] studied the ovarian sensitivity measuring the proportion of follicles growing to a certain extent and calculated the Follicular Output Rate (FORT) [28]. They found that, in non-overweight PCOS women [27], there was a statistically increased number of recruited follicles upon triggering, but in overweight PCOS, this was only confirmed for follicles above 14 mm of diameter as well as for FORT [28]. Other outcomes are detailed in Table 2.

#### 3.2.2. Endocrine Profile and Premature LH Surge

Regarding endocrine profiles, Jiang et al. [20] observed increased LH and progesterone levels at triggering in the PPOS + LE protocol compared to PPOS alone, with non-a significant difference in estradiol levels. Liu et al. [27], in the PPOS + LE protocol, describes significantly lower estradiol levels and higher LH levels at each observation point during COS and significantly increased progesterone levels from day 9 of COS until the triggering day and the day after. Furthermore, in high-BMI PCOS women, Liu et al. [28] confirmed significantly lower estradiol levels at each observation point during COS in the PPOS + LE protocol, as well as significantly increased LH levels at triggering and significantly increased progesterone levels at day 9–11. Wang et al. [29] showed increased starting-day levels of estradiol even after PSM, and also increased LH levels and decreased estradiol levels at triggering in the PPOS + LE protocol. Last, Wang et al. [30] demonstrated that for both POSEIDON group 3 and 4, LH levels were higher and estradiol levels were lower on the triggering day.

#### 3.2.3. Impact on Pregnancy Outcomes

In women with normal ovarian reserve, Jiang et al. [20] found a statistically increased biochemical pregnancy rate after frozen embryo transfer in the PPOS + LE protocol and a trend towards increased clinical pregnancy and implantation rates, while no differences were noted for miscarriage and live birth rates. Liu et al. [27] observed significantly higher implantation rates in PCOS women, independently from different endometrial preparations, but no differences were seen for miscarriage rates. Wang et al. [29] showed significantly increased clinical pregnancy rates, but not live birth rates. Nonetheless, Wang et al. [30] in POSEIDON group 3 and 4 and Liu et al. [28] in high-BMI PCOS women did not find differences in pregnancy outcomes.

## 4. Discussion

The addition of letrozole to gonadotrophin in PPOS protocol increases the number of retrieved and mature oocytes in the general IVF population, normal ovarian reserve women, and the POSEIDON group 4, but not in PCOS women or POSEIDON group 3. Moreover, it extends the duration of COS and reduces the amount of drugs (gonadotrophins and eventually MPA) in the general IVF population and normal ovarian reserve women, but not in PCOS or POSEIDON group 3 and 4 women. Checking the hormone levels, all the studies observed increased levels of LH and progesterone in the PPOS + LE group, with lower levels of estradiol. Nonetheless, in terms of pregnancy outcomes, although some studies did not find differences, it was demonstrated that biochemical, implantation, and clinical pregnancy rates after frozen embryo transfer can be ameliorated by the use of letrozole during COS. The primary strength of this review lies in its rigorous methods, as well as in its novelty and the specific clinical question it seeks to address. It offers the first comprehensive overview of a promising protocol change, providing clinicians with an update on the current knowledge. However, this novelty is also inherently connected to its main limitation: the evidence is still developing and relies entirely on retrospective cohort studies. Such studies are naturally prone to selection bias, confounding factors, and information bias, all of which can impact the validity of their findings. In addition, only nine studies (including the excluded ones) have been published to date on the association between letrozole and PPOS in controlled ovarian stimulation, of which five were included in our systematic review; this is a relatively small number to draw definitive conclusions. While some included studies employed strong statistical techniques, such as propensity score matching, to minimize these biases, they cannot match the methodological rigor of a prospective, randomized controlled trial (RCT). Moreover, the included populations only come from China and, at the same time, there is significant population heterogeneity, varying from general IVF and normal ovarian reserve to PCOS and poor responders as POSEIDON groups, thus limiting the generalizability of the results. Furthermore, there is considerable variation in the interventions as well. The PPOS protocols lacked consistency across different studies. The daily dose of MPA, the most commonly used progestin, ranged from 4 to 10 milligrams and was modified according to LH levels during COS. This dose-dependent variation in the suppressive agent directly affects pituitary inhibition, a key aspect targeted by letrozole co-treatment. Therefore, a quantitative meta-analysis was deemed unsuitable and was not performed. This decision was based on the significant clinical and methodological differences among the included studies, which would render any pooled estimate statistically invalid and potentially misleading in a clinical context. The main implication for these results is that letrozole could give the advantage of reduced cost (reduction in drug consumption), even though the stimulation might last longer, maintaining a comparable or even greater retrieval of oocyte and rate of mature eggs, as well as comparable and sometimes even better pregnancy outcomes. The literature indicates that the PPOS protocol aims to prevent premature LH surges, making it a safe and effective approach while letrozole optimizes follicular recruitment. This combination is particularly beneficial for poor responders, where every recruited follicle maximizes the chances of oocyte retrieval. Furthermore, letrozole can promote more homogeneous growth of the follicular cohort, reducing the follicular asynchrony often observed in patients with a diminished ovarian reserve [35]. Adding letrozole to PPOS alone does not intend to replace PPOS, but aims to enhance and refine it by addressing its primary physiological limitation: significant pituitary suppression.

Increased levels of LH and progesterone during COS with letrozole supplementation were already found during the luteal phase [36] and also during the late follicular phase [37,38]. Initially, a low level of estradiol and its reduced negative feedback on the pituitary indirectly increase gonadotrophins’ secretion (FSH and LH). Regarding the mechanism through which LE elevates progesterone, it was hypothesized [39] that the accumulation of androgens (e.g., testosterone and androstenedione), induced by the inhibition of estrogen production by LE [38] causes accumulation of progesterone when a certain threshold is reached [37,38]. Indeed, in PPOS cycles, the eventual detrimental effects of progesterone on the endometrium are not of clinical importance, given the freeze-all strategy. Possible effects on the oocyte depend also on the number of retrieved ones, since it is believed that the number of mature follicles has a role in the evaluation of progesterone levels; in fact, when the number of oocytes is high, like in high-responders, this does not compromise pregnancy outcomes [40].

Nonetheless, LH is crucial for final oocyte maturation, and depriving follicular growth of LH can impair the oocyte’s cytoplasmic competence. Suppressing the pituitary and LH leads to inadequate follicular maturation, angiogenesis, and cell proliferation [41,42]. This energy deficiency hampers the function of the meiotic spindle, which is essential for proper chromosome segregation during meiosis [43,44]. Spindle defects are a primary cause of chromosomal non-disjunction, resulting in aneuploidy, where the chromosome count is abnormal. This risk may be higher in women of advanced reproductive age, increasing the likelihood of aneuploidy, as happens for POSEIDON 4 [45,46]. For instance, letrozole may be beneficial by inhibiting the conversion of androgens into estrogens, thus stimulating higher endogenous gonadotropin production without harming oocyte quality. Although some evidence suggests that letrozole might impair oocyte maturity, existing fertility preservation studies indicate that it is safe and does not reduce the number of mature oocytes (metaphase II, MII) retrieved compared to standard stimulation protocols [47]. However, in women with LH deficiency, the addition of rLH to rFSH improves live birth rates over rFSH alone [48,49,50]. To move from an experimental to a more established approach, large-scale, multicenter, prospective, randomized controlled trials or bidirectional analyses of prospectively collected data are necessary to confirm its value in clinical practice. A standardized progestin protocol is essential to prevent bias selection.

## 5. Conclusions

The combined use of letrozole and gonadotrophins within the PPOS protocol shows promise as an innovative approach for controlled ovarian stimulation. Although current findings are from retrospective studies, they suggest that incorporating letrozole effectively counteracts the intense pituitary suppression typically associated with standard PPOS, increasing LH levels and the number of retrieved and mature oocytes in normal and poor responders, but not in PCOS women. Indeed, retrospective data do not allow for definitive conclusions to be drawn and, therefore, further studies, and eventually RCTs, are needed to confirm these results on different ethnic populations and to assess if there is a specific infertile subgroup that could take more advantage of this protocol. Ongoing research should focus on refining the protocol, including experimenting with less suppressive progestins to make ovarian stimulation safer, more effective, and more physiological for all patients.

## Figures and Tables

**Table 1:** General characteristics of the included studies.

Authors, Year	Country	Study Period and Design	Population	Intervention	Comparison	Outcome	Results
Jiang et al., 2022 [20]	Shiyan, China	October 2017–2019, Retrospective Cohort	576 normal ovarian reserve	Group A: 327 PPOS + LE	Group B: 249 PPOS alone	Oocyte recruited and mature; days of gonadotrophins; total dose of gonadotrophins, total dose of MPA, levels of LH, FSH, progesterone and estradiol; fertilization rate, cleavage rate, blastocyst formation rate, high-quality embryo rate; methods of embryo preparation; stage of embryo transfer; endometrial thickness; number of embryos transferred; biochemical pregnancy rate, CPR, implantation rate, miscarriage rate, ectopic pregnancy rate and LBR.	Days of gonadotrophins were less in PPOS + LE; lower dose of MPA and gonadotrophins for stimulation in PPOS + LE; higher number of retrieved and mature oocytes in PPOS + LE; higher fertilization rate, cleavage rate, and blastocyst formation rate in PPOS + LE; similar pregnancy outcome.
Liu et al., 2022 [27]	Shanghai, China	January 2018–March 2021, Retrospective Cohort	448 PCOS normal BMI	Group A: 224 PPOS + LE	Group B: 224 PPOS alone	Oocyte recruited and mature and fertilized; retrieval, mature and fertilization rate; duration of stimulation; total dose of gonadotrophins, levels of LH, FSH, progesterone and estradiol during stimulation; fertilization rate, cleavage stage and rate, blastocyst stage, high-quality embryo, cryopreserved rate, viable embryos; number of follicles > 10 mm at triggering; number of follicles > 14 mm at triggering; endometrial preparation; endometrial thickness; cancelation rate for nonviable embryos; OHSS; premature LH surge; cumulative CPR per transfer, implantation rate, miscarriage rate, ectopic pregnancy rate.	The implantation rate was higher in PPOS + LE; Letrozole co-treatment was associated with decreased oocyte maturity and fertilization rates compared to PPOS alone; higher number of follicles > 10 and >14 mm at triggering in PPOS + LE; lower percentage of women with profound pituitary suppression in PPOS + LE. Lower levels of estradiol and higher levels of LH and progesterone during stimulation in PPOS + LE.
Liu et al., 2023 [28]	Shanghai, China	January 2017–September 2022, Retrospective Cohort	268 PCOS high BMI	Group A: 134 PPOS + LE	Group B: 134 PPOS alone	Oocytes retrieved, mature oocytes, fertilized oocytes, cleaved embryos, blastocyst embryos, high-quality embryos, viable embryos, cryopreserved embryos; cycle cancelation rate; FSH, LH, estradiol and progesterone levels during stimulation; mature oocyte rate, oocyte retrieval rate, fertilization rate; cleaved embryo rate; aspirated follicle number; FORT, follicles 10–12 mm at triggering, follicles 12–14 mm at triggering, follicles 14–16 mm at triggering, follicles > 16 mm at triggering; hMG dose, duration of stimulation; embryo transfer stage; number of embryo transferred; endometrial preparation and thickness, implantation rate, clinical pregnancy rate, miscarriage rate, LBR per cycle and per patient, ectopic pregnancy rate; neonatal weight.	Higher number of follicles 14–16 mm and >16 mm at triggering and higher FORT in PPOS + LE; lower mature oocyte rate in PPOS + LE; lower levels of estradiol, higher levels of LH during stimulation, progesterone increased only D9-11 during stimulation in PPOS + LE.
Wang et al., 2024 [29]	Shiyan, China	January 2016–2019, Retrospective Cohort	2575 cycles (general infertile women)	Group A: 379 PPOS + LE	Group B: 379 PPOS alone	FSH, LH, estradiol at starting and at triggering during stimulation; duration of stimulation; dose of MPA and dose of gonadotrophins; retrieved and mature oocytes; number of fertilizations; good quality embryos; clinical pregnancy rate and live birth rate; stage of embryo transfer, number of embryos transferred.	The numbers of oocytes retrieved, mature oocytes, fertilization, and clinical pregnancy rates were more favorable in the PPOS + LE group than in the PPOS group. Duration of stimulation was more, dose of MPA was lower, LH was higher and estradiol was lower at triggering in PPOS + LE.
Wang et al., 2025 [30]	Shanghai, China	January 2018–2021, Retrospective Cohort	557 POSEIDON: Group 3 (189) and Group 4 (368)	Group A: 111 POSEIDON 3; Group B: 214 POSEIDON 4 PPOS + LE	Group A: 78 Poseidon 3; Group B 154 Poseidon 4 PPOS alone	Total dose of gonadotrophins; duration of stimulation; LH and estradiol levels at triggering; Retrieved oocytes, MII oocytes, two pronuclei (2PN) embryos, available embryos (for cryopreservation), oocyte maturation rate (MII oocytes/total retrieved oocytes), normal fertilization rate (2PN embryos/MII oocytes), available embryo rate (available embryo/2PN cleavage embryos), premature LH surge rate. Endometrial thickness, endometrial preparation; dominant follicle; number of embryos transferred, stage of embryo at transfer. cancelation rate; ongoing pregnancy rate, biochemical pregnancy rate, clinical pregnancy rate, miscarriage rate, live birth rate, cumulative LBR and cumulative CPR; gestational age at delivery, neonatal gender and weight, delivery mode, neonatal and maternal complications.	Estradiol at triggering was lower and LH at triggering higher both for POSEIDON 3 and 4 in PPOS + LE; For POSEIDON group 4, the number of oocytes retrieved, mature oocytes, 2PN embryos and number of available embryos was higher in the PPOS + LE group. Gestational age at delivery was longer and mean birthweight was higher in PPOS + LE.

BMI: Body Mass Index; CPR: Clinical Pregnancy Rate; FORT: Follicular Output Rate; FSH: follicle stimulating hormone; hMG: Human Menopausal Gonadotropin; LBR: Live Birth Rate; LE: Letrozole; LH: luteinizing hormone; MII: metaphase II; MPA: Medroxy Progesterone Acetate; OHSS: Ovarian Hyperstimulation Syndrome; PCOS: PolyCystic Ovary Syndrome; POSEIDON: Patient-Oriented Strategies Encompassing IndividualizeD Oocyte Number; PPOS: Progestin-Primed Ovarian stimulation.

**Table 2:** Details of controlled ovarian stimulation.

Authors, Year	ART	Gonadotrophin	Dose	Trigger	Progesterone Dose	Letrozole Dose	Day of Administration	Transfer
Jiang et al., 2022 [20]	IVF/ICSI	r-FSH	75–150 IU (PPOS + LE); 150–225 IU (PPOS)	0.1 mg triptorelin and 2000 IU hCG	MPA (10 mg)	2.5 mg	LE: D2 of the menstrual cycle; MPA: gradually reduced according to serum LH level	256 GnRHa FET HRT or FET HRT (PPOS + LE); 225 GnRHa FET HRT or FET HRT (PPOS); DET or SET; D3 or D5 ET
Liu et al., 2022 [27]	IVF/ICSI	hMG	150–225 IU	0.1 mg triptorelin and 1000 IU hCG	MPA (4 mg/d)	2.5 mg	LE: D3-D7; MPA: D3	280 FET HRT (PPOS + LE); 294 FET HRT (PPOS); SET; D3 or D5 ET
Liu et al., 2023 [28]	IVF/ICSI	hMG	150–225 IU	0.1 mg triptorelin and 1000 IU hCG	MPA (4 mg/d)	2.5 mg	LE: D3-D7; MPA: D3	163 FET HRT (PPOS + LE); 193 FET HRT (PPOS); SET; D3 or D5 ET
Wang et al., 2024 [29]	IVF/ICSI	Not stated	Not stated	0.1 mg triptorelin and 2000 IU hCG	MPA (dose not specified)	dose not specified	LE: D3-D7; MPA: D6	GnRHa FET HRT; SET; D5 ET
Wang et al., 2025 [30]	IVF/ICSI	Not stated	150–300 IU	0.1 mg triptorelin and 10,000 IU hCG OR 10,000 IU hCG OR 250 mgc r-hCG	MPA (10 mg/d)	2.5–5 mg	LE: D2-D6; MPA: D2 until day trigger	GnRHa FET HRT or FET HRT; SET; D5 ET

ART: Assisted Reproductive Technology; DET: Double Embryo Transfer; ET: Embryo Transfer; FET: Frozen Embryo Transfer; GnRHa: Gonadotropin-Releasing Hormone Agonist; hCG: Human Chorionic Gonadotropin; hMG: Human Menopausal Gonadotropin; HRT: Hormone Replacement Therapy; ICSI: Intracytoplasmic Sperm Injection; IU: International Unit; IVF: In Vitro Fertilization; LE: Letrozole; MPA: Medroxyprogesterone Acetate; PPOS: Progestin-Primed Ovarian Stimulation; r-FSH: Recombinant Follicle-Stimulating Hormone; r-hCG: Recombinant Human Chorionic Gonadotropin; SET: Single Embryo Transfer.

## Data Availability

The original contributions presented in the study are included in the article, further inquiries can be directed to the corresponding authors.

## Supplementary Materials

The following supporting information can be downloaded at https://www.mdpi.com/article/10.3390/jcm15020410/s1; Supplementary Materials Table S1. PRISMA checklist for systematic review; Supplementary Materials Table S2. Quality assessment of included studies.

## References

[1] Grisendi V., La Marca A. (2017). Individualization of controlled ovarian stimulation in vitro fertilization using ovarian reserve markers. Minerva Ginecol..

[2] Sunkara S.K., Rittenberg V., Raine-Fenning N., Bhattacharya S., Zamora J., Coomarasamy A. (2011). Association between the number of eggs and live birth in IVF treatment: An analysis of 400,135 treatment cycles. Hum. Reprod..

[3] Harvey A.J., Willson B.E., Surrey E.S., Gardner D.K. (2025). Ovarian stimulation protocols: Impact on oocyte and endometrial quality and function. Fertil. Steril..

[4] Glujovsky D., Pesce R., Miguens M., Sueldo C.E., Lattes K., Ciapponi A. (2020). How effective are the non-conventional ovarian stimulation protocols in ART? A systematic review and meta-analysis. J. Assist. Reprod. Genet..

[5] Evans N.P., Richter T.A., Skinner D.C., Robinson J.E. (2002). Neuroendocrine mechanisms underlying the effects of progesterone on the oestradiol-induced GnRH/LH surge. Reprod. Suppl..

[6] Chabbert-Buffeta N., Skinner D.C., Caraty A., Bouchard P. (2000). Neuroendocrine effects of progesterone. Steroids.

[7] Giles J., Cruz F., Garcia-Velasco J.A. (2024). Progestin-primed ovarian stimulation. Curr. Opin. Obstet. Gynecol..

[8] Iorio G.G., Rovetto M.Y., Conforti A., Carbone L., Vallone R., Cariati F., Bagnulo F., Di Girolamo R., La Marca A., Alviggi C. (2021). Severe Ovarian Hyperstimulation Syndrome in a Woman with Breast Cancer Under Letrozole Triggered with GnRH Agonist: A Case Report and Review of the Literature. Front. Reprod. Health.

[9] Iorio G.G., Carbone L., Conforti A., Rovetto M.Y., Picarelli S., Cariati F., Strina I., Papanikolaou E., Alviggi C. (2022). Ovarian Hyperstimulation Syndrome after GnRH Agonist Triggering and Freeze-All Protocol? Never Not, Hardly Ever: A Systematic Review of Case Reports. Gynecol. Obstet. Investig..

[10] Vaiarelli A., Pittana E., Cimadomo D., Ruffa A., Colamaria S., Argento C., Giuliani M., Petrone P., Fabozzi G., Innocenti F. (2025). A multicycle approach through DuoStim with a progestin-primed ovarian stimulation (PPOS) protocol: A valuable option in poor prognosis patients undergoing PGT-A. J. Assist. Reprod. Genet..

[11] Haas J., Casper R.F. (2017). In vitro fertilization treatments with the use of clomiphene citrate or letrozole. Fertil. Steril..

[12] Andersen C.Y., Lossl K. (2008). Increased intrafollicular androgen levels affect human granulosa cell secretion of anti-Mullerian hormone and inhibin-B. Fertil. Steril..

[13] Nielsen M.E., Rasmussen I.A., Kristensen S.G., Christensen S.T., Møllgård K., Wreford Andersen E., Byskov A.G., Yding Andersen C. (2011). In human granulosa cells from small antral follicles, androgen receptor mRNA and androgen levels in follicular fluid correlate with FSH receptor mRNA. Mol. Hum. Reprod..

[14] Casper R.F., Mitwally M.F. (2011). Use of the aromatase inhibitor letrozole for ovulation induction in women with polycystic ovarian syndrome. Clin. Obstet. Gynecol..

[15] Vendola K., Zhou J., Wang J., Famuyiwa O.A., Bievre M., Bondy C.A. (1999). Androgens promote oocyte insulin-like growth factor I expression and initiation of follicle development in the primate ovary. Biol. Reprod..

[16] Lossl K., Freiesleben N.L.C., Wissing M.L., Petersen K.B., Holt M.D., Mamsen L.S., Anderson R.A., Andersen C.Y. (2020). Biological and clinical rationale for androgen priming in ovarian stimulation. Front. Endocrinol..

[17] Gallo A., Di Spiezio Sardo A., Conforti A., Iorio G.G., Zizolfi B., Buonfantino C., De Angelis M.C., Strina I., Marrone V., Bifulco G. (2024). Assessing ovarian stimulation with letrozole and levonorgestrel intrauterine system after combined fertility-sparing approach for atypical endometrial lesions: A retrospective case-control study. Reprod. Biomed. Online.

[18] Mukherjee A.G., Wanjari U.R., Nagarajan D., Vibhaa K.K., Anagha V., Joshua Paul P., Tharani Priya T., Chakraborty R., Renu K., Dey A. (2022). Letrozole: Pharmacology, toxicity, and potential therapeutic effects. Life Sci..

[19] Lalami I., Labrosse J., Cedrin-Durnerin I., Comtet M., Vinolas C., Krief F., Sifer C., Peigne M., Grynberg M. (2022). Is letrozole during ovarian stimulation useful in breast cancer patients undergoing fertility preservation to reduce early luteal progesterone levels following GnRH-agonist trigger?. Reprod. Biol. Endocrinol..

[20] Jiang X., Jiang S., Diao H., Deng K., Zhang C. (2022). Progestin-primed ovarian stimulation protocol with or without letrozole for patients with normal ovarian reserve: A retrospective cohort study. J. Clin. Pharm. Ther..

[21] Page M.J., McKenzie J.E., Bossuyt P.M., Boutron I., Hoffmann T.C., Mulrow C.D., Shamseer L., Tetzlaff J.M., Akl E.A., Brennan S.E. (2021). The PRISMA 2020 statement: An updated guideline for reporting systematic reviews. BMJ.

[22] Arce J.C., Nyboe Andersen A., Collins J. (2005). Resolving methodological and clinical issues in the design of efficacy trials in assisted reproductive technologies: A mini-review. Hum. Reprod..

[23] PICO Search Strategy. https://casp-uk.net/news/pico-search-strategy/.

[24] Zegers-Hochschild F., Adamson G.D., Dyer S., Racowsky C., de Mouzon J., Sokol R., Rienzi L., Sunde A., Schmidt L., Cooke I.D. (2017). The International Glossary on Infertility and Fertility Care, 2017. Fertil. Steril..

[25] Stang A. (2010). Critical evaluation of the Newcastle-Ottawa scale for the assessment of the quality of nonrandomized studies in meta-analyses. Eur. J. Epidemiol..

[26] Cook D.A., Reed D.A. (2015). Appraising the quality of medical education research methods: The Medical Education Research Study Quality Instrument and the Newcastle-Ottawa Scale-Education. Acad. Med..

[27] Liu Y., Lin J., Chen L., Mao X., Wang L., Chen Q., Yu S., Kuang Y. (2022). Letrozole cotreatment with progestin-primed ovarian stimulation in women with polycystic ovary syndrome undergoing IVF treatment. Front. Physiol..

[28] Liu Y., Lin J., Shen X., Zhu Q., Kuang Y. (2023). Letrozole cotreatment improves the follicular output rate in high-body-mass-index women with polycystic ovary syndrome undergoing IVF treatment. Front. Endocrinol..

[29] Wang X., Zhang Y., Diao H., Jiang S., Zhang C. (2024). Letrozole cotreatment progestin-primed ovarian stimulation in women undergoing controlled ovarian stimulation for in vitro fertilization. J. Obstet. Gynaecol. Res..

[30] Wang L., Xie J., Chu Y., Chen J., Jin L., Yue J. (2025). Effect of letrozole cotreatment in progestin-primed ovarian stimulation on IVF/ICSI outcomes in POSEIDON group 3 and 4 poor responders: A retrospective cohort study. Eur. J. Med. Res..

[31] Li H.L., Shen B.B., He Z.L., Wang H.L., Sun Z.F. (2024). Progestin-primed ovarian stimulation with letrozole using different doses of medroxyprogesterone acetate per day: A retrospective cohort study. Front. Endocrinol..

[32] Wang X., Tian J., Tian L., Chen X., Zhang Z., Diao H., Zhang Y. (2025). The Follicular Output Rate was Improved with 3-Day Letrozole Administration Compared with 5-Day Letrozole Administration Under Progestin-Primed Ovarian Stimulation. Drug Des. Devel. Ther..

[33] Zhang Y., Li H., Zhu S., Jiang S., Zhao W., Wang X., Tian L., Zhao G., He N., Diao H. (2023). The comparison between fixed versus degressive doses of medroxyprogesterone acetate combined with letrozole in patients of progestin-primed ovarian stimulation protocol: A propensity score-matched study. Front. Endocrinol..

[34] Li X., Chang Y. (2021). Clinical application of letrozole combined with progestin-primed ovarian stimulation protocol in poor ovarian responders at advanced age. Fertil. Steril..

[35] Guan S., Feng Y., Huang Y., Huang J. (2021). Progestin-Primed Ovarian Stimulation Protocol for Patients in Assisted Reproductive Technology: A Meta-Analysis of Randomized Controlled Trials. Front. Endocrinol..

[36] Alviggi C., Marci R., Vallone R., Conforti A., Di Rella F., Strina I., Picarelli S., De Rosa P., De Laurentiis M., Yding Andersen C. (2017). High progesterone levels during the luteal phase related to the use of an aromatase inhibitor in breast cancer patients. Eur. Rev. Med. Pharmacol. Sci..

[37] Yang X., Lin G., Lu G., Gong F. (2019). Letrozole supplementation during controlled ovarian stimulation in expected high responders: A pilot randomized controlled study. Reprod. Biol. Endocrinol..

[38] Bülow N.S., Skouby S.O., Warzecha A.K., Udengaard H., Andersen C.Y., Holt M.D., Grøndahl M.L., Nyboe Andersen A., Sopa N., Mikkelsen A.L.E. (2022). Impact of letrozole co-treatment during ovarian stimulation with gonadotrophins for IVF: A multicentre, randomized, double-blinded placebo-controlled trial. Hum. Reprod..

[39] Liu R., Zhou L., Chen X., He H., Cai Z. (2022). Letrozole Supplementation and the Increased Risk of Elevated Progesterone Levels on Trigger Day. Front. Endocrinol..

[40] Griesinger G., Mannaerts B., Andersen C.Y., Witjes H., Kolibianakis E.M., Gordon K. (2013). Progesterone elevation does not compromise pregnancy rates in high responders: A pooled analysis of in vitro fertilization patients treated with recombinant follicle-stimulating hormone/gonadotropin-releasing hormone antagonist in six trials. Fertil. Steril..

[41] Conforti A., Di Girolamo R., Guida M., Alviggi C., Casarini L. (2025). Pharmacogenomics of LH and its receptor: Are we ready for clinical practice?. Reprod. Biol. Endocrinol..

[42] Conforti A., Tüttelmann F., Alviggi C., Behre H.M., Fischer R., Hu L., Polyzos N.P., Chuderland D., Rama Raju G.A., D’Hooghe T. (2022). Effect of Genetic Variants of Gonadotropins and Their Receptors on Ovarian Stimulation Outcomes: A Delphi Consensus. Front. Endocrinol..

[43] Strączyńska P., Papis K., Morawiec E., Czerwiński M., Gajewski Z., Olejek A., Bednarska-Czerwińska A. (2022). Signaling mechanisms and their regulation during in vivo or in vitro maturation of mammalian oocytes. Reprod. Biol. Endocrinol..

[44] MacLennan M., Crichton J.H., Playfoot C.J., Adams I.R. (2015). Oocyte development, meiosis, and aneuploidy. Semin. Cell Dev. Biol..

[45] Elmerdahl Frederiksen L., Ølgaard S.M., Roos L., Petersen O.B., Rode L., Hartwig T., Ekelund C.K., Vogel I., Danish Central Cytogenetics Registry Study Group (2024). Maternal age and the risk of fetal aneuploidy: A nationwide cohort study of more than 500 000 singleton pregnancies in Denmark from 2008 to 2017. Acta Obstet. Gynecol. Scand..

[46] Esteves S.C., Yarali H., Ubaldi F.M., Carvalho J.F., Bento F.C., Vaiarelli A., Cimadomo D., Özbek İ.Y., Polat M., Bozdag G. (2020). Validation of ART Calculator for Predicting the Number of Metaphase II Oocytes Required for Obtaining at Least One Euploid Blastocyst for Transfer in Couples Undergoing in vitro Fertilization/Intracytoplasmic Sperm Injection. Front. Endocrinol..

[47] Sonigo C., Sermondade N., Calvo J., Benard J., Sifer C., Grynberg M. (2019). Impact of letrozole supplementation during ovarian stimulation for fertility preservation in breast cancer patients. Eur. J. Obstet. Gynecol. Reprod. Biol. X.

[48] Conforti A., Esteves S.C., Humaidan P., Longobardi S., D’Hooghe T., Orvieto R., Vaiarelli A., Cimadomo D., Rienzi L., Ubaldi F.M. (2021). Recombinant human luteinizing hormone co-treatment in ovarian stimulation for assisted reproductive technology in women of advanced reproductive age: A systematic review and meta-analysis of randomized controlled trials. Reprod. Biol. Endocrinol..

[49] Marchiani S., Tamburrino L., Benini F., Pallecchi M., Bignozzi C., Conforti A., Alviggi C., Vignozzi L., Danza G., Pellegrini S. (2020). LH supplementation of ovarian stimulation protocols influences follicular fluid steroid composition contributing to the improvement of ovarian response in poor responder women. Sci. Rep..

[50] Conforti A., Carbone L., Di Girolamo R., Iorio G.G., Guida M., Campitiello M.R., Ubaldi F.M., Rienzi L., Vaiarelli A., Cimadomo D. (2025). Therapeutic management in women with a diminished ovarian reserve: A systematic review and meta-analysis of randomized controlled trials. Fertil. Steril..

