# Supplementary material for: Letrozole Co-Administration in Progestin-Primed Ovarian Stimulation (PPOS) Protocols for Patients Undergoing In Vitro Fertilization: A Systematic Review
Source: J Clin Med. 2026 Jan 6;15(2):410. doi: 10.3390/jcm15020410 (PMC12842092; doi:10.3390/jcm15020410)
Supplement: Supplementary file 1 [file jcm-15-00410-s001.zip › Supplementary Table S2.pdf]

Supplementary Table S2. Quality assessment of included studies.

Authors	Selection	Comparability	Outcome
Jiang et Al., 2021 [20]	★★	★	★★
Liu et Al., 2022 [27]	★★	★★	★★
Liu et Al., 2023 [28]	★★	★★	★★
Wang et Al., 2024 [29]	★★	★	★
Wang et Al., 2025 [30]	★★	★	★★
